# Reversible Fluindione-Induced Chronic Interstitial Nephritis

**DOI:** 10.1155/2016/9818195

**Published:** 2016-04-05

**Authors:** Thomas Crepin, Jamal Bamoulid, Cécile Courivaud, Omar Dahmani, Sophie Felix, Didier Ducloux

**Affiliations:** ^1^Department of Nephrology, Dialysis, and Renal Transplantation, CHU Besançon, 25030 Besançon, France; ^2^Department of Nephrology and Dialysis, CH Saint Claude, 39200 Saint-Claude, France; ^3^Department of Histopathology, CHU Besançon, 25030 Besançon, France

## Abstract

Fluindione is well known to induce acute drug-induced interstitial nephritis (IN). Most cases occurred soon after the onset of treatment. We report a unique case of severe subacute fluindione-induced IN diagnosed 2 years after the treatment was begun. Renal function dramatically improved after fluindione withdrawal and steroid therapy.

## 1. Introduction

In biopsy series, IN accounts for 6.5–27% of acute kidney injuries. Drugs are the more frequent cause of IN and are involved in nearly 70% of cases of acute IN in adults [1, 2]. Most frequent drugs involved are antimicrobial agents and nonsteroidal anti-inflammatory drugs [1, 3]. Drug-induced IN usually presents as acute renal failure associated or not with allergic symptoms. In most cases, symptoms occur early and diagnosis is made soon after the start of the drug. We report here an unusual case of drug-induced IN diagnosed and successfully treated more than two years after the initiation of the drug.

## 2. Case Report

Mr. B., aged 81, was referred to our unit because of renal failure. He had a past history of thrombophlebitis with pulmonary embolism that occurred 4 and 2 years ago, respectively. He was treated with fluindione (Previscan®) since the last episode. Other antecedents were chronic bronchitis to wood dust,* Helicobacter pylori* positive chronic gastritis 10 years ago, and total hip replacement 6 years ago. He did not receive any drug except fluindione. He did not take any self-medication.

Blood pressure was 130/80 mmHg and heart rate 75/min. Physical examination was normal.

Blood electrolytes were normal. Serum creatinine was 298 *μ*mol/L. He displayed aregenerative anemia (80 mg/dL) without hypereosinophilia. Electrophoresis of serum proteins showed polyclonal hypergammaglobulinemia, moderate hypoalbuminemia (30 g/L, normal: 39.7–49.4 g/L), high fibrinogen level (5.75 g/L, normal: 2–4 g/L), and normal C reactive protein level (3 mg/L, normal: <6 mg/L). Angiotensin converting enzyme was 16 nmol/mL/mn (normal: 12–25 nmol/mL/mn). There was neither hematuria nor leukocyturia and urinary protein excretion was 1 g/24 h. Immunological tests were negative. Renal ultrasound depicted two kidneys with normal size (11 centimeters), well differentiated and without obstruction on urinary tract.

Two years earlier, during hospitalization for pulmonary embolism, serum creatinine level was 61 *μ*mol/L.

**Figure 1:**
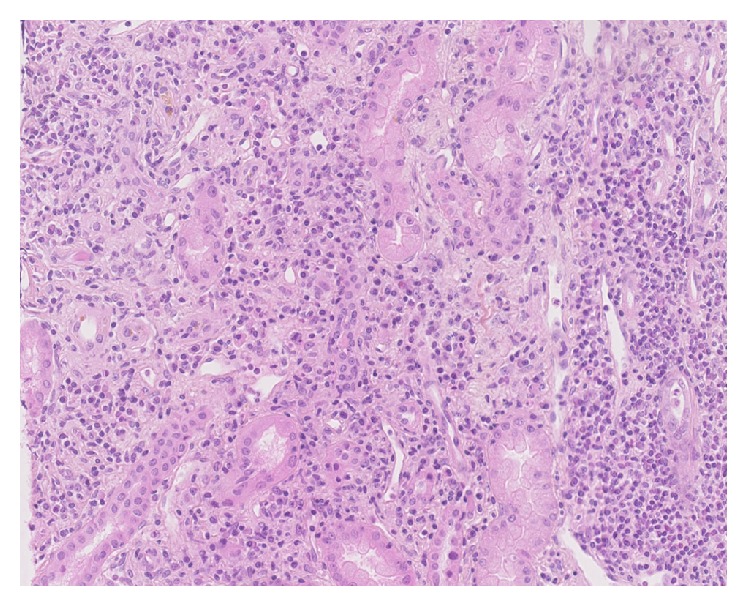
Kidney biopsy. Optical microscopy showed significant interstitial lymphocytic infiltration (HES).

**Figure 2:**
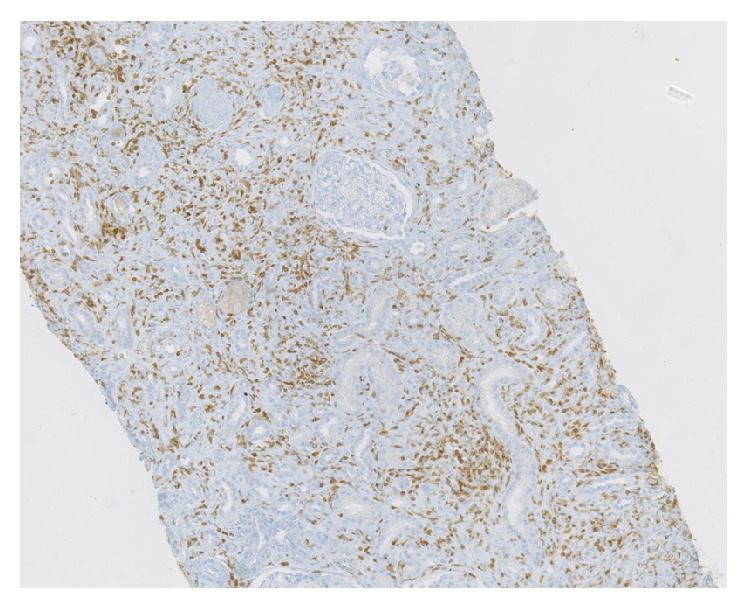
Kidney biopsy. Optical microscopy showed significant interstitial CD3^+^ infiltration (immune-staining CD3 polyclonal, Dako, 1/100).

Kidney biopsy was performed. Optical microscopy showed tubulointerstitial nephritis with marked inflammation T cell CD3^+^, few eosinophils, and severe fibrosis with tubular atrophy. Immunofluorescent studies did not reveal any immune deposits (Figures 1-2).

Fluindione was switched to warfarin (Coumadin®). One month later, we observed a decrease in serum creatinine to 210 *μ*mol/L. Nevertheless, steroid therapy was started at 0.5 mg/kg equivalent prednisone during a period of two months with progressive tapering. At the end of the treatment, serum creatinine had dramatically improved to 100 *μ*mol/L. No adverse effects of steroid were noted.

## 3. Discussion

We reported a case of late-diagnosed fluindione-induced interstitial nephritis (IN). Histological examination showed interstitial nephritis compatible with the diagnosis. The patient did not receive any other treatment. No other causes were identified. Finally, renal function improved after fluindione withdrawal and normalized after steroid therapy. All these arguments strongly plead for the diagnosis of fluindione-induced IN.

Only few case reports or small series (*n* = 1 to 24) of fluindione-induced IN have been published [4–8]. Considering the large diffusion of this drug, the prevalence of fluindione-induced interstitial nephritis is probably low. Nevertheless, underreporting or underdiagnosis cannot be excluded. Mean delay between the start of fluindione and symptom is 12 ± 7 weeks (range: 3–28). Renal disease is often severe with mean serum creatinine at diagnosis of 460 ± 265 *μ*mol/L. In our case, diagnosis was made after two years of treatment. The patient did not display any allergic symptoms that could have enabled more prompt diagnosis. Interestingly, renal failure was reversible even when fluindione exposition has been long and associated with severe histological lesions. Indeed, Cam et al. reported that fluindione-induced IN often have an unfavorable evolution to severe chronic renal failure and chronic dialysis. Recently, Leven et al. [9] showed in a retrospective study that 33% of drug-induced IN are induced by vitamin K antagonists and in nearly 90% of cases by fluindione. Two cases of drug-induced IN to warfarin have indeed been described [8, 10]. Average time for the occurrence of fluindione-induced IN is usually found in the literature to be 9.75 ± 5.85 weeks (range: 4–22). Leven et al. confirm the severity of renal disease in fluindione-induced IN with persistent highly impaired renal function at 3 months for 87% of patients, half with chronic kidney disease stage IV or V.

Drug withdrawal is the cornerstone of treatment of drug-induced interstitial nephritis that can itself allow an improvement in renal function. Steroids are much debated. Their effectiveness is variable depending on the data of Cam et al. where 84% of patients received corticosteroid therapy without clear improvement in renal function. Nevertheless, benefit seems greater when steroids are begun soon after diagnosis (<15 days) and in the absence of significant fibrosis and tubular atrophy on renal histology [1, 3, 11]. Corticosteroid therapy is prescribed in a dose between 0.5 and 1 mg/Kg over a period of 2 to 6 weeks but there is no specific protocol or recommendation.

## 4. Conclusion

In conclusion, fluindione should be suspected and discontinued with interstitial nephritis regardless of the time between drug introduction and diagnosis of kidney failure. Renal function should be regularly monitored in patients under fluindione treatment even after 28 weeks of treatment. If there is a preexisting chronic kidney disease, warfarin should be the first-line choice due to the risk of non-*ad integrum* recovery renal function in fluindione-induced IN. In view of these adverse effects, the appropriateness of the use of fluindione compared to coumarin derivatives remains to be discussed.
